# Retrospective evaluation of the effects of pulmonary artery and aortic diameters on hospitalization duration and survival in patients hospitalized with COVID-19

**DOI:** 10.5578/tt.202402930

**Published:** 2024-06-12

**Authors:** Zarifa ABDULLAYEVA

**Affiliations:** 1 Clinic of Pulmonology, Etlik City Hospital, Ankara, Türkiye; 2 Clinic of Pulmonology, Health Sciences University Faculty of Medicine, Gülhane Education and Research Hospital, Ankara, Türkiye; 3 Clinic of Radiology, Health Sciences University Faculty of Medicine, Gülhane Education and Research Hospital, Ankara, Türkiye

## Abstract

**ABSTRACT**

**
Retrospective evaluation of the effects of pulmonary artery and aortic diameters on hospitalization duration and survival in patients hospitalized with COVID-19
**

**Introduction:** *This study explores the impact of vascular diameters on mortality risk in Coronavirus disease-2019 (COVID-19) patients. COVID-19, caused by severe acute respiratory syndrome Coronavirus 2 (SARS-CoV-2), presents diverse clinical manifestations and is associated with thrombosis.
*

**Materials and Methods:** *In this study, we retrospectively examined the data of patients who were hospitalized and treated in our hospital between September 1, 2020, and November 30, 2020, and whose COVID-19 diagnosis was confirmed by reverse transcriptase polymerase chain reaction (RT-PCR). The diameters of the ascending aorta, main pulmonary artery, and right and left pulmonary arteries were measured from the chest computed tomography (CT) scans taken at the time of admission. The aim of the study was to inves- tigate the impact of vascular diameters on the course of the disease.
*

**Results:** *Of 1.705 patients, 840 were eligible for the study. We concluded that 36 of the patients (4.3%) died, and among the non-survivors patients, 12 (33.3%) were females, and 24 (66.7%) were males. Hospitalization duration was 7.1 ± 3.1 vs. 6.1 ± 2 days (p= 0.004) in surviving and non-surviving patients respectively. On the other hand, we found the mean diameters of the right pulmonary artery in the chest CT of patients to be 2.17 ± 0.35 vs. 2.44
*

*
± 0.29 cm in survivors and non-survivors, respectively (p< 0.001). In addition, we found the mean diameters of the left pulmonary artery 2.12 ± 0.32 vs.
*

*
2.34 ± 0.28 cm in survivors and non-survivors, respectively (p< 0.001). Mean diameters of the ascending aorta were 3.53 ± 0.46 vs. 3.72 ± 0.34 cm in survivors and non-survivors, respectively (p= 0.017).
*

**Conclusion:** *The study underscores the potential prognostic value of vascular diameters, especially in the ascending aorta and main pulmonary artery, as
*

*
indicators of mortality risk in COVID-19 patients. The association between vascular dilation and severity of COVID-19, coupled with elevated D-dimer levels, suggests a link between thrombosis and vascular involvement.
*

**Key words:** *COVID-19; SARS-CoV-2; ascending aorta; pulmonary artery; death
*

**ÖZ**

**
COVID-19 tanısı ile hospitalize edilen hastalarda pulmoner arter ve aort çaplarının hastane yatış süresi ve sağkalım üzerine etkilerinin retrospektif olarak değerlendirilmesi
**

**Giriş:** *Bu çalışma, Koronavirüs hastalığı-2019 (COVID-19) hastalarında damar çaplarının mortalite riski üzerindeki etkisini araştırmak- tadır. Şiddetli akut solunum sendromu Koronavirüs 2 (SARS-CoV-2)’nin neden olduğu COVID-19, çeşitli klinik belirtiler ve tromboz ile ilişkilidir.
*

**Materyal ve Metod:** *Bu çalışmada 1 Eylül 2020 ile 30 Kasım 2020 tarihleri arasında hastanemizde yatarak tedavi gören ve ters trans- kriptaz polimeraz zincir reaksiyonu (RT-PCR) ile COVID-19 tanısı doğrulanan hastaların verileri geriye dönük olarak incelendi. Başvuru anında çekilen akciğer bilgisayarlı tomografisinden (BT) çıkan aort, ana pulmoner arter ile sağ ve sol pulmoner arter çapları ölçüldü. Çalışmanın amacı damar çaplarının hastalığın seyrine etkisini araştırmaktır.
*

**Bulgular:** *Çalışmaya 1705 hastanın 840’ı uygun bulundu. Hastalardan 36 (%4,3)’sının öldüğü, ölen hastaların ise 12 (%33,3)’sinin kadın, 24 (%66,7)’ünün erkek olduğu görüldü. Hastanede kalış süresi yaşayan ve ölen hastalarda sırasıyla 7,1 ± 3,1 gün’e karşılık 6,1 ± 2 gün (p= 0,004) idi. Diğer yandan hastaların akciğer BT’sinde sağ pulmoner arter çapını sırasıyla yaşayan ve ölen hastalarda 2,17 ± 0,35 cm’e karşılık 2,44 ± 0,29 cm olarak bulunmuştur (p< 0,001). Ayrıca sol pulmoner arter çapını sırasıyla yaşayan ve ölen hastalarda 2,12 ± 0,32 cm’ye karşılık 2,34 ± 0,28 cm olarak bulunmuştur (p< 0,001). Ortalama çıkan aort çapı ise sırasıyla yaşayan ve ölen hastalarda 3,53 ± 0,46 cm’ye karşılık 3,72 ± 0,34 cm idi (p= 0,017).
*

**Sonuç:** *Çalışma, COVID-19 hastalarında mortalite riskinin bir göstergesi olarak özellikle çıkan aort ve ana pulmoner arterdeki çapların potansiyel prognostik değerinin altını çizmektedir. Vasküler genişleme ile COVID-19'un ciddiyeti arasındaki ilişki, yüksek D-dimer seviyeleriyle birleştiğinde, tromboz ile vasküler tutulum arasında bir bağlantı olduğunu düşündürmektedir.
*

**Anahtar kelimeler:** *COVID-19; SARS-CoV-2; asendan aort; pulmoner arter; ölüm
*

## INTRODUCTION

Coronavirus disease-2019 (COVID-19) is an infec- tious disease caused by the acute respiratory syn- drome Coronavirus-2 (SARS-CoV-2) virus that belongs to the family Coronaviridae. It manifests with differ- ent clinical presentations from an asymptomatic car- rier to severe pneumonia with hypoxemia (1).

Risk factors of COVID-19 include advanced age, male sex, hypertension, diabetes, chronic renal failure, cardiovascular disease, chronic lung disease, and immunosuppression status (2,3). The gold standard diagnostic test for COVID-19 is the real-time polymerase chain reaction (RT-PCR). Although the test has low sensitivity, its specificity is high (4). Radiological findings of the lung may also aid in the diagnosis of COVID-19 (5).

High pressure in pulmonary arteries is associated with pulmonary vascular resistance. It has been shown that there is an increased risk of thrombosis and mortality in COVID-19 (6). Lung dissection materials revealed that the patients had vasculitis and small pulmonary vessel occlusion findings (7).

Although the disease might affect almost any organ, previous studies have reported that the most lethal

form of the disease is characterized by lung involvement and a tendency to develop vascular thrombosis. The autopsy series also showed that thrombosis develops in the pulmonary vascular bed and the entire vascular system of the body (8). It has been shown that increased pulmonary artery diameters in diseases that involve the pulmonary vascular system are associated with poor prognosis (9). It has also been reported that pulmonary artery diameter might be associated with mortality in patients with COVID-19 (10). This study aimed to determine the effects of the diameters of the ascending aorta, main pulmonary artery, and right and left pulmonary arteries during the disease in patients whose COVID-19 diagnosis was confirmed with RT-PCR and who were hospitalized and received treatment.

## MATERIALS and METHODS

The study was conducted with the data obtained from patients who were followed up with a diagnosis of COVID-19 at Gülhane Training and Research Hospital between September 1, 2020, and November 30, 2020. The study was approved by the Scientific Research Ethics Committee of the University Gülhane Training and Research Hospital (approval number

46418926, meeting number 2021–05, and project

number 2021–100).

Inclusion criteria were as follows: Patients >18 years old with COVID-19 diagnosis proven using the RT-PCR test, and whose thorax computed tomography (CT) was performed within the first 24 hours of admission. Exclusion criteria were as follows: Patients who were immediately admitted to the intensive care unit upon their arrival at the hospital, those diagnosed with aortic aneurysm or pulmonary hypertension, those diagnosed with pulmonary embolism within the last six months, those with duration of hospital stay <four days, and those whose data was inaccessible.

Demographic characteristics (e.g. age and sex), laboratory parameters, final clinical condition, thorax CT scans, and duration of hospital stay were collected from the patient’s medical records. The initial values of the biochemical parameters obtained upon admission, including the levels of C-reactive protein (CRP), troponin I, ferritin, fibrinogen, D-dimer, interleukin-6 (IL-6), and lymphocyte parameters were utilized for this study.

Thorax CT images were obtained without the use of a contrast medium on a 64-row CT device (Aquillon

64, Toshiba, Japan). All the images were evaluated by a thoracic radiologist with at least 20 years of experience. The diameters of the main pulmonary artery, the tubular level of the ascending aorta, and the right and left main pulmonary arteries were measured (in cm) using CT by selecting the best motionless phase from the widest part within the proximal 1 cm (Figure 1).

The patients were divided into two groups (survivors and non-survivors) according to their final clinical condition. Biochemical parameters, especially the diameters of the ascending aorta and pulmonary vascular structures, demographic characteristics, and duration of hospital stay were compared between the groups.

## Statistical Analysis

The data obtained in the study was transferred to a computer and Microsoft Office Excel 2016 was used for the initial data entry. Statistical analysis of the data was performed with SPSS for Mac Version 20.00 (SPSS Inc., Chicago, IL., United States of America) package. The compliance of continuous variables with the normal distribution was evaluated using the Kolmogorov–Smirnov test. Continuous variables with normal distribution were presented as mean ±

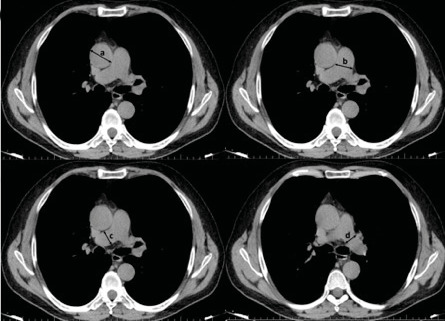

**Figure 1.** Measurement of vascular structures diameter on axial thorax computed tomography slices

ascending aorta **(A)**. Main pulmonary artery **(B)**. Right pulmonary artery **(C)**. Left pulmonary artery **(D)**.

standard deviation and median (minimum–maximum) for non-normal variables. The Mann-Whitney U test or Student’s *t*-test was used to evaluate the differences between the groups, depending on whether it was a normal distribution or not, and a p< 0.05 was considered statistically significant.

## RESULTS

A retrospective analysis was conducted on the data collected from 1.705 patients followed up with a diagnosis of COVID-19 at Gülhane Training and Research Hospital between September 1, 2020, and November 30, 2020. Of these, 686 patients who failed to meet the inclusion criteria and 179 who met the exclusion criteria were excluded from the study. Finally, the data obtained from 840 patients were evaluated in the present study. The study flowchart is given in Figure 2.

Mean age of the patients in the study population was

61.9 ± 15.5 years, 362 (43.1%) of them were women, 478 (56.9%) were men, and their mean age was 63.7

± 15.7 vs. 60.7 ± 15.2 years, respectively. Upon admission, the biochemical parameters are follows: Median CRP= 84.3 (0.3–410.1) mg/L and D-dimer=

0.76 (0.2–80) mg/L; ascending aorta and main

pulmonary artery diameters measured using thorax CT= 3.54 ± 0.45 cm and 2.73 ± 0.4 cm, respectively

(Table 1).

Considering the final clinical condition, 36 (4.3%) of the 840 patients died. Of the non-survivors, 12 (33.3%) were women and 24 (66.7%) were men. It was also noted that age was higher in non-survivors compared with the survivors (73.5 ± 12.6 vs. 61.4 ±

15.4 years). A significant difference was observed in the levels of troponin, fibrinogen, D-dimer, and IL-6 between the two groups (Table 2).

Non-survivors had larger diameters of the vascular structures compared with those of the survivors (Table 3). A notable finding was the relationship between the vascular diameters and the D-dimer values. D-dimer value was measured in 818 patients at the time of admission, and 66.9% (n= 562) of them had elevated D-dimer levels (>0.5 mg/L). Patients with elevated D-dimer levels exhibited significantly larger vascular diameters. The right PA diameter was found to be 2.17 ± 0.35 vs. 2.44 ± 0.29 cm (p< 0.001) between the survivors and non-survivors, respectively, and the left PA diameter was 2.12 ± 0.32 vs. 2.34 ± 0.28 cm (p< 0.001) between the survivors and non-survivors, respectively.

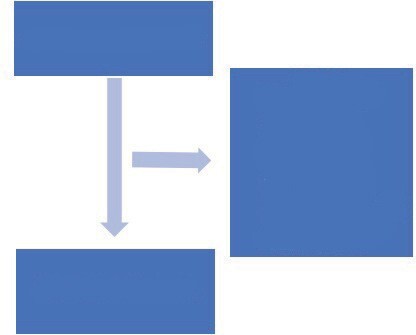

**Figure 2.** Flowchart of study patients.

**Table:** 

**Table 1.** Characteristics of the study population	
**Variables**	**Mean ± SD / Median (min-max)**
Age, years	61.9 ± 15.5
Male, n (%)	478 (56.9%)
Female, n (%)	362 (43.1%)
CRP, mg/L	84.3 (0.3-410.1)
Troponin, ng/mL	7.28 (0.9-1723)
Ferritin, ng/mL	223.5 (4.2-4784)
Fibrinogen, mg/dL	520 (37-900)
D-dimer, mg/L	0.76 (0.2-80)
IL-6, pg/mL	30.3 (0-1620)
Lymphocyte, x103	1.1 (0.1-63.8)
Ascending aorta, cm	3.54 ± 0.45
Main PA, cm	2.73 ± 0.40
Right PA, cm	2.18 ± 0.36
Left PA, cm	2.13 ± 0.32
CRP: C-reactive protein, IL-6: Interleukin 6, PA: Pulmonary artery, SD: Standard deviation.	

**Table:** 

**Table 2.** Comparison of demographic and clinical features of the survivors and non-survivors			
**Variables**	**Survivors (n= 804)**	**Non-survivors (n= 36)**	**p**
Age, years	61.4 ± 15.4	73.5 ± 12.6	**<0.001**
Sex/male, n (%)	454 (56.5)	24 (66.6)	0.227
Length of stay, days	7.1 ± 3.1	6.1 ± 2	**0.004**
CRP, mg/L	82.3 (0.3-410.1)	95.4 (0.4-278.1)	0.090
Troponin, ng/mL	7.1 (0.9-1723(	13.4 (1.6-860.7)	**0.001**
Ferritin, ng/mL	224.8 (4.2-4784)	167.4 (9.5-1500)	0.122
Fibrinogen, mg/dL	522 (149-900)	453.5 (37-815)	**0.007**
D-dimer, mg/L	0.75 (0.2-80)	1.33 (0.3-26.9)	**0.011**
IL-6, pg/mL	29.8 (0-1620)	54.9 (0.1-197.8)	**0.027**
Lymphocyte, x103	1.1 (0.1-40.5)	0.9 (0.2-63.8)	0.056
CRP: C-reactive protein, IL-6: Interleukin 6. Bold fonts indicate statistically significante p-values.			

**Table:** 

**Table 3.** Differences in vascular diameter between the survivors and non-survivors			
**Vascular structures**	**Survivors (n= 804)**	**Non-survivors (n= 36)**	**p**
Ascending aorta, cm	3.53 ± 0.46	3.72 ± 0.34	**0.017**
Main PA, cm	2.73 ± 0.4	2.83 ± 0.37	0.155
Main PA/Aa ratio	0.77 (0.4-1.28)	0.76 (0.5-0.98)	0.463
Right PA, cm	2.17 ± 0.35	2.44 ± 0.29	**<0.001**
Left PA, cm	2.12 ± 0.32	2.34 ± 0.28	**<0.001**
PA: Pulmonary artery, Aa: Ascending aorta. Bold fonts indicate statistically signifacante p-values.			

## DISCUSSION

In the present study, we determined the effects of the diameters of the ascending aorta, main pulmonary artery, and right and left pulmonary arteries on mortality rate and duration of hospital stay in patients whose COVID-19 diagnosis was proven with RT-PCR and who were hospitalized for treatment and follow- up.

The increase in the diameters of the main vascular structures significantly increased the risk of mortality. Moreover, advanced age and male sex significantly increased the risk of mortality in patients hospitalized with a diagnosis of COVID-19. It was shown that COVID-19 increases the risk of thrombosis that is associated with mortality (6,11). Increased serum levels of D-dimer, a fibrin degradation product, are an indicator of thrombosis. In patients with COVID- 19, D-dimer levels may remain elevated depending on the severity of the disease and risk of mortality (12). It is believed that the increased D-dimer levels are associated with the formation of numerous microthrombi in many body systems. In our patient population, D-dimer levels were high at 1.84 ± 5.42 mg/L and were significantly higher in non-survivors. Furthermore, elevated D-dimer levels were significantly associated with the risk of mortality.

Mean diameters of the ascending aorta and main pulmonary artery are 3.2 ± 0.4 cm and 2.51 ± 0.28 cm, respectively in the normal population, and these values increase with cardiopulmonary pathologies (13,14). Mean diameters of the ascending aorta, main pulmonary artery, and the right and left pulmonary arteries were 3.54 ± 0.45 cm, 2.73 ± 0.4 cm, and

2.18 ± 0.36 cm and 2.13 ± 0.32 cm, respectively in the present study population. The evaluation of the relationship between the vascular diameters and the course of the disease revealed that the diameters of the ascending aorta, main pulmonary artery, and right and left pulmonary arteries significantly increased in patients hospitalized with COVID-19 diagnosis, thereby contributing to an increased risk of mortality. An increased pressure in the pulmonary arteries is associated with pulmonary vascular resistance. The published autopsy results on COVID-19 showed the development of vascular congestion and thrombi in small vessels and capillaries (8). Vasculitis and small pulmonary vessel occlusion were observed in the lung dissection samples (7). Autopsy studies revealed thrombotic microangiopathy in the lung, small vessel thrombus formation in the periphery of the lung,

mostly associated with alveolar hemorrhage foci and the presence of microthrombi in the pulmonary capillaries (8). A prospective cohort study by Wichmann et al. on 12 patients who died of COVID-

19 and underwent autopsy showed that the most common histopathological findings were widespread alveolar damage, microvascular thromboembolism, capillary congestion, and protein-rich interstitial edema in the lungs (15). These findings indicate an increase in the resistance of the pulmonary vascular bed, possibly causing increased pressure and dilation in the pulmonary arteries. In contrast, the disease usually manifests itself with hypercoagulability and is associated with an elevated incidence of pulmonary thrombotic complications, causing dysfunction by increasing the afterload of the right ventricle (16). Eslami et al. have reported that the widespread lung involvement due to COVID-19 was positively associated with the increased diameter of the main pulmonary artery (17). D’Andrea et al. have reported that the mean pulmonary artery pressure increased in patients with COVID-19 pneumonia which was associated with an elevated risk of hospital mortality (18). Similarly, a significant relationship between pulmonary artery diameter and mortality in COVID- 19 pneumonia was also reported (19).

Pulmonary vascular resistance increases in acute pulmonary thromboembolism because of mechanical occlusion of the vascular bed with thrombus and hypoxic vasoconstriction of the pulmonary arterial system. Vasoconstriction induced through mediators, such as thromboxane A2 and serotonin, contributed to the current manifestation (20). It has been reported that the cumulative incidence of pulmonary embolism in COVID-19 infection was 24% (50% in patients hospitalized in the intensive care units and 18% in other patients) (21,22). A possible explanation for the dilatation in the pulmonary arteries of these patients is acute pulmonary embolism or increased pulmonary artery pressure because of microthrombi in the peripheral pulmonary branches. However, as our patient population was evaluated with non-contrast CT scans, we were unable to determine the existence of a thrombus in the pulmonary arteries.

A significant increase in the radiological prevalence of lung parenchymal areas and pulmonary artery diameters was observed in COVID-19 pneumonia, indicating that pulmonary vascular diameters might be used to monitor pulmonary hypertension because of lung damage and to predict the disease prognosis (19).

The increased pulmonary artery diameter, which is an indicator of pulmonary hypertension secondary to lung fibrosis, is expected to occur in the long term. However, an acute expansion in the diameter of the pulmonary artery may also occur because of lung damage. As mentioned previously, dilatation was observed in the pulmonary arteries within days in cases where acute respiratory distress syndrome was imminent (23). One of the most important limitations of the present study was that lung parenchyma evaluation was not performed in the present study population.

The evaluation of the diameter of the ascending aorta showed that it was significantly larger in the non- survivors compared with the survivors. Hypertension was common in hospitalized patients with COVID-

19 pneumonia owing to the complication of myocardial damage caused by the infection. It has also been shown that abnormal ascending aorta and left ventricle enlargement are associated with severe inflammation and heart damage (24). Interstitial mononuclear inflammatory infiltrates and epicardial edema findings were identified in the heart tissue on conducting the postmortem analysis of a patient with COVID-19 (25).

We know that our study has limitations. Its most important limitation is that it is a retrospective and single-center study. In addition, other limitations include the lack of previous echocardiography and/ or pulmonary CT examinations of the patients, the fact that the thorax CT scans were non-contrast, and the fact that parenchymal involvement was not evaluated in the evaluation. We think that the high number of cases and the fact that all evaluations were made by the same radiologist are important aspects of our study.

## CONCLUSION

It is already known that advanced age and male sex are contributing risk factors for mortality in COVID- 19 infection. The diameters of the ascending aorta, main pulmonary artery, and right and left pulmonary arteries may be associated with the prediction of the severity of the disease and mortality in hospitalized patients.

**Ethical Committee Approval:** This study was approved by Health Science University Faculty of Medicine Clinical Research Committee (Decision no: 2021/100 Date: 11.03.2021).

## CONFLICT of INTEREST

The authors declare that they have no conflict of interest.

## AUTHORSHIP CONTRIBUTIONS

Concept/Design: DD, CT, YA Analys/Interpretation: DD Data acqusition: DD, NÖ Writing: DD, UB

Clinical Revision: CT, UB Final Approval: DD

## REFERENCES

1 *
  Guan W, Ni Z, Hu Y, Liang W, Ou C, He J, et al. Clinical characteristics of coronavirus disease 2019 in China. N Engl J Med 2020; 382(18): 1708-20. https://doi.org/10.1056/NEJMoa2002032*
2 *
  Zhou F, Yu T, Du R, Fan G, Liu Y, Liu Z, et al. Clinical course and risk factors for mortality of adult inpatients with COVID-19 in Wuhan, China: A retrospective cohort study. Lancet 2020; 395(10229): 1054-62. https://doi.org/10.1016/S0140-6736(20)30566-3*
3 *
  Petrilli CM, Jones SA, Yang J, Rajagopalan H, O’Donnell L, Chernyak Y. et al. Factors associated with hospital admis- sion and critical illness among 5279 people with corona- virus disease 2019 in New York City: Prospective cohort study. BMJ 2020; 369: m1966. https://doi.org/10.1136/bmj.m1966*
4 *
  He JL, Luo L, Luo ZD, Lyu JX, Ng MY, Shen XP. et al. Diagnostic performance between CT and initial real-time RT-PCR for clinically suspected 2019 coronavirus disease (COVID-19) patients outside Wuhan, China. Respir Med 2020; 168: 105980. https://doi.org/10.1016/j.rmed.2020.105980*
5 *
  Doğan D, Cüce F, Akay S, Ayaz T, Kınık D, Savaşçı Ü, et al. Normal chest CT prevalence in coronavirus disease 2019 (COVID-19) Patients: A report of 791 cases. Acta Med 2019: 5(32); 2917-21.
  *
6 *
  Lodigiani C, Iapichino G, Carenzo L, Cecconi M, Ferrazzi P, Sebastian T, et al. Venous and arterial thromboembolic complications in COVID-19 patients admitted to an aca- demic hospital in Milan, Italy. Thromb Res 2020; 191: 9-14. https://doi.org/10.1016/j.thromres.2020.04.024*
7 *
  Luo W, Yu H, Gou J, Li X, SunY, Li J, et al. Clinical patholo- gy of critical patients with novel coronavirus pneumonia (COVID-19). Preprints 2020; 2020020407.
  *
8 *
  Fox SE, Akmatbekov A, Harbert JL, Li G, Brown JQ, Vander Heide RS. Pulmonary and cardiac pathology in African American patients with COVID-19: An autopsy series from New Orleans. Lancet Respir Med 2020; 8(7): 681-6. https://doi.org/10.1016/S2213-2600(20)30243-5*
9 *
  Shen Y, Wan C, Tian P, Wu Y, Li X, Yang T, et al. CT-Base pulmonary artery measurement in the detection of pulmo- nary hypertension. Medicine (Baltimore) 2014; 93: e256. https://doi.org/10.1097/MD.0000000000000256*
10 *
  Spagnolo P, Cozzi A, Foà RA, Spinazzola A, Monfardini L, Bnà C, et al. CT-derived pulmonary vascular metrics and clinical outcome in COVID-19 patients. Quant Imaging Med Surg 2020; 10(6): 1325-33. https://doi.org/10.21037/qims-20-546*
11 *
  Arslan Y, Yılmaz G, Doğan D, Hasırcı M, Çetindoğan H, Öcal N, et al. The effectiveness of early anticoagulant treatment in Covid-19 patients. Phlebology 2021; 36(5): 384-91. https://doi.org/10.1177/0268355520975595*
12 *
  Wells PS, Anderson DR, Rodger M, Forgie M, Kearon C, Dreyer J, et al. Evaluation of D-dimer in the diagnosis of suspected deep-vein thrombosis. N Engl J Med 2003; 349(13): 1227-35. https://doi.org/10.1056/NEJMoa023153*
13 *
  Paruchuri V, Salhab KF, Kuzmik G, Gubernikoff G, Fang H, Rizzo JA, et al. Aortic size distribution in the general pop- ulation: Explaining the size paradox in aortic dissection. Cardiology 2015; 131(4): 265-72. https://doi.org/10.1159/000381281*
14 *
  Truong QA, Massaro JM, Rogers IS, Mahabadi AA, Kriegel MF, Fox CS, et al. Reference values for normal pulmonary artery dimensions by non-contrast cardiac computed tomography: The framingham heart study. Circ Cardiovasc Imaging 2012; 5(1): 147-54. https://doi.org/10.1161/CIRCIMAGING.111.968610*
15 *
  Wichmann D, Sperhake JP, Lütgehetmann M, Steurer S, Edler C, Heinemann A, et al. Autopsy findings and venous thromboembolism in patients with COVID-19: A prospec- tive cohort study. Ann Intern Med 2020; 173(4): 268-77. https://doi.org/10.7326/M20-2003*
16 *
  Lippi G, Sanchis-Gomar F, Favaloro EJ, Lavie CJ, Henry BM. Coronavirus disease 2019-associated coagulopathy. Mayo Clin Proc 2021; 96(1): 203-17. https://doi.org/10.1016/j.mayocp.2020.10.031*
17 *
  Eslami V, Abrishami A, Zarei E, Khalili N, Baharvand Z, Sanei-Taheri M. The association of CT-measured cardiac indices with lung involvement and clinical outcome in patients with COVID-19. Acad Radiol 2021; 28(1): 8-17. https://doi.org/10.1016/j.acra.2020.09.012*
18 *
  D’Andrea A, Scarafile R, Riegler L, Liccardo B, Crescibene F, Cocchia R, et al. Right ventricular function and pulmo- nary pressures as independent predictors of survival in patients with COVID-19 pneumonia. JACC Cardiovasc Imaging 2020; 13(11): 2467-8. https://doi.org/10.1016/j.jcmg.2020.06.004*
19 *
  Raoufi M, Safavi Naini SAA, Azizan Z, Jafar Zade F, Shojaeian F, Ghanbari Boroujeni M, et al. Correlation between chest computed tomography scan findings and mortality of COVID-19 cases; A cross-sectional study. Arch Acad Emerg Med 2020; 8(1): e57.
  *
20 *
  Smulders YM. Pathophysiology and treatment of hemody- namic instability in acute pulmonary embolism: The piv- otal role of pulmonary vasoconstriction. Cardiovasc Res 2000; 48: 23-33. https://doi.org/10.1016/S0008-6363(00)00168-1*
21 *
  Chen, J., Wang, X., Zhang, S., Lin, B., Wu, X., Wang, Y., et al. Characteristics of acute pulmonary embolism in patients with COVID-19 associated pneumonia from the city of Wuhan. Clinical and Applied Thrombosis/ Hemostasis, 2020:26, https://doi.org/1076029620936772.
  *
22 *
  Bompard F, Monnier H, Saab I, Tordjman M, Abdoul H, Fournier L, et al. Pulmonary embolism in patients with COVID-19 pneumonia. Eur Respir J 2020; 56(1): 2001365. https://doi.org/10.1183/13993003.01365-2020*
23 *
  Price LC, McAuley DF, Marino PS, Finney SJ, Griffiths MJ, Wort SJ. Pathophysiology of pulmonary hypertension in acute lung injury. Am J Physiol Lung Cell Mol Physiol 2012; 302(9): L803-15. https://doi.org/10.1152/ajplung.00355.2011*
24 *
  Song L, Zhao S, Wang L, Yang K, Xiao W, Clifford S, et al. Cardiovascular changes in patients with COVID-19 from Wuhan, China. Front Cardiovasc Med 2020; 7: 150. https://doi.org/10.3389/fcvm.2020.00150*
25 *
  Liu X, Wang R, Qu G, Wang Y, Liu P, Zhu Y, et al. Histological findings of COVID‐19 based on autopsy: A case report. J Forensic Med 2020; 36; 1-3.
  *

